# In-situ high-resolution visualization of laser-induced periodic nanostructures driven by optical feedback

**DOI:** 10.1038/s41598-017-16646-1

**Published:** 2017-11-28

**Authors:** Alberto Aguilar, Cyril Mauclair, Nicolas Faure, Jean-Philippe Colombier, Razvan Stoian

**Affiliations:** Laboratoire Hubert Curien, UMR 5516 CNRS, Université de Lyon, Université Jean Monnet, 42000 St. Etienne, France

## Abstract

Optical feedback is often evoked in laser-induced periodic nanostructures. Visualizing the coupling between surfaces and light requires highly-resolved imaging methods. We propose *in*-*situ* structured-illumination-microscopy to observe ultrafast-laser-induced nanostructures during fabrication on metallic glass surfaces. This resolves the pulse-to-pulse development of periodic structures on a single irradiation site and indicates the optical feedback on surface topographies. Firstly, the quasi-constancy of the ripples pattern and the reinforcement of the surface relief with the same spatial positioning indicates a phase-locking mechanism that stabilizes and amplifies the ordered corrugation. Secondly, on sites with uncorrelated initial corrugation, we observe ripple patterns spatially in-phase. These feedback aspects rely on the electromagnetic interplay between the laser pulse and the surface relief, stabilizing the pattern in period and position. They are critically dependent on the space-time coherence of the exciting pulse. This suggests a modulation of energy according to the topography of the surface with a pattern phase imposed by the driving pulse. A scattering and interference model for ripple formation on surfaces supports the experimental observations. This relies on self-phase-stabilized far-field interaction between surface scattered wavelets and the incoming pulse front.

## Introduction

The interaction of ultrafast pulsed laser radiation with materials in ablative regimes is at the origin of spectacular material structures with characteristic sizes beyond the diffraction limit at the incident wavelength^1^. These fabricated features are then able to add a range of novel properties to the engineered material. From a process control perspective, the fundamental challenge stays with defining those light and material characteristics able to facilitate structuring of matter on mesoscopic scales. The controlled and reproducible fabrication of laser-induced structures significantly smaller than the processing optical wavelengths has thus the potential to open up the way towards efficient laser-based nanostructuring strategies. In this perspective, the ability to go on sub-micron dimensions in a controlled manner is critical for the development of laser nanotechnologies and for the design of multiscale structured materials. New surface functions can be obtained from combining structures sizes into hierarchical ensambles mixing micron and nanoscale features. These functions span a large application field, from optical and surface color design^2,3^ to controlled hydrophobicity^4–6^, hydrophilicity^7^ and bio-mimetic properties^8,9^. Besides the fabrication aspect and in a perspective of enforcing control, the challenge consists of visualising structures with the highest achievable accuracy^10^. The need to monitor the fabrication process in a rapid manner makes optical technologies of interest. A tremendous effort was made recently in interrogating structures and objects with optical means giving access to the nanoscale. To overcome the diffraction limit in optical microscopy, a variety of techniques have been developed preserving at the same time the advantages of non contact investigations^11^. For instance, Stimulated Emission Depletion (STED) microscopy, a super-resolution technique based on functional groups of molecules that show fluorescence can achieve a lateral resolution of 20 nm^12^. Also, stochastic super resolution techniques such as Stochastic Optical Reconstruction Microscopy (STORM) and Photoactivated Localization Microscopy (PALM) can be used to overcome optical diffraction limit with similar performance^13–15^. These developments together with the present laser processing techniques enable an increased ability to produce and observe at the same time laser-induced structures at and below the diffraction limit, during fabrication, improving the understanding and facilitating the control of the structuring process.

The nonlinear interaction involving ultrafast laser radiation can confine the energy on spatial scales below 100 nm via direct focusing^1,16^. This represents a localized absorption event that can structure matter beyond the diffraction limit. The ultimate size of the modification is then defined mainly by the material response to energy absorption and transport, with scales significantly smaller than those related to light confinement. This response can be of thermomechanical, hydrodynamic, or ablative nature^1,17,18^. One of the most spectacular laser nanostructuring processes is the formation of sub-wavelength ripples on materials ranging from metals to dielectrics. Specifically, laser radiation can trigger periodic arrangements of matter with periods going down to *λ*/5*n*, with *λ* being the processing wavelength and *n* the material optical index, (see refs^19,20^ and references therein) in spite of spatially smooth, slowly-varying incident optical fields. The so-called laser-induced periodic structures (LIPSS)^21^ combine nonlinear interaction with matter destabilization and light effects on surface topographies and are dynamically driven by optical feedback. This represents an intricate relation between the field, surface topography and its growth dynamics, where the coupling will spatially shape laser fields and local domains of energy absorption into the material. The definition of local and nonlocal collective interactions are key to the development of nanoprocessing control strategies on larger areas^22^. Thus, the understanding of the behavior of dynamical surfaces and the evolving coupling between topography and light is of paramount importance in upgrading the ripples arrangement quality, but equally in providing the physical insights behind material periodic rearrangement. Visualizing the process *in*-*situ* with resolutions capable of resolving the arrangement periods is therefore key to grasping the material dynamics and its feedback-driven topography. In the context of periodic arrangements, the sub-wavelength *λ*/*n* spaced low-spatial-frequency LIPSS (LSFL) carry a particular interest for developing patterning methods that can render particular optical, mechanical and tactile functions. Their periodicities in the range of 500–700 nm (for the typical 800 nm processing wavelength) can create optical effects in the visible domain and morphology effects on liquid contact angles on surfaces. Therefore, there is a particular interest in observing *in*-*situ* how the optical response of a real surface in the context of ripple formation can be drastically influenced by the surface morphology and its evolution towards ordered corrugation.

We discuss here using *in*-*situ* spatially-resolved observations the development of LSFL on metallic glass surfaces on a single site, from the pristine surface to the full development of the ripple pattern. The material choice is motivated the quality of ripples formation. The LIPSS alignment, which suffers often from periodicity errors and bifurcations believed to originate from self-organizational dynamics^23^, has a particular quality in laser irradiated metallic glasses^24^. In the ripple development we indicate the action range of two types of optical feedback; one related to surface topography (scattering and localization at inhomogeneities) and the second determined by the self-driven coherent interactions between driving and scattered light at constant relative phase. We apply a super-resolution optical method based on structured illumination microscopy (SIM) to observe and reconstruct LIPSS behavior during multipulse formation with *≈*100 nm accuracy. This allows to clearly define the topography role in establishing and growing rippled patterns. The optical resolution allows for a very high quality in the optical imaging with resolving features not observable in standard optical microscopy. The *in*-*situ* implementation of the technique permits to observe the gradual development of regular structures from rough surfaces under multipulse exposure on the same site and allows to put forward a mechanism that stabilizes the pattern spatially. By analyzing the evolving relief on the single site, an accurate analysis of the role of light coupling on complex topographies and pattern locking mechanisms is conducted. We demonstrate intrinsic phase-locking stabilizing patterns and periodicities during ripples growth that is related to surface corrugation and light scattering. The technique allows equally to define a second feedback that can phase-lock and stabilize the pattern from independent random sites. The mechanism is intrinsically related to the self-interference between the driving and scattered light at constant relative phase. We believe that this approach carries a significant potential to observe features that are essential for nanostructures formation, that were only elusively indicated before. The understanding of periodic arrangement is essential for strategies of large area patterning using laser-based technologies.

## Results and Discussion

### High-resolution microscopy observation

A Zr_41.2_Ti_13.8_Cu_12.5_Ni_10_Be_25.5_ (atom%) metallic superalloy undercooled to a glassy structure (bulk metallic glass, BMG)^25,26^ is used for laser nanostructuring, in the conditions where its thermoplasticity above the glass transition temperature can render high quality LIPSS^24,27^. Single-side polished Zr-BMG samples (Ra < 20 nm) with a diameter of 8 mm and a thickness of 3 mm were used in the experiment. Polishing was done using diamond paste and alumina powder with grit size down to 0.1 *μ*m. The sample, positioned on high precision XYZ stages, was then subject to ultrafast laser irradiation derived from an amplified Ti:Sapphire laser system delivering 120 fs laser pulses at a nominal repetition rate of 1 kHz and 800 nm incident wavelength. Single pulse where selected using electro-optical and mechanical shutters and are impinging on the surface along the normal direction. The irradiation geometry follows a common path with the observation tool, as described in the Methods section, with a rather tight focusing. For each pulse, the *in*-*situ* SIM-picture acquisition was performed in order to follow the pulse-to pulse LIPSS growth.

**Figure 1:**
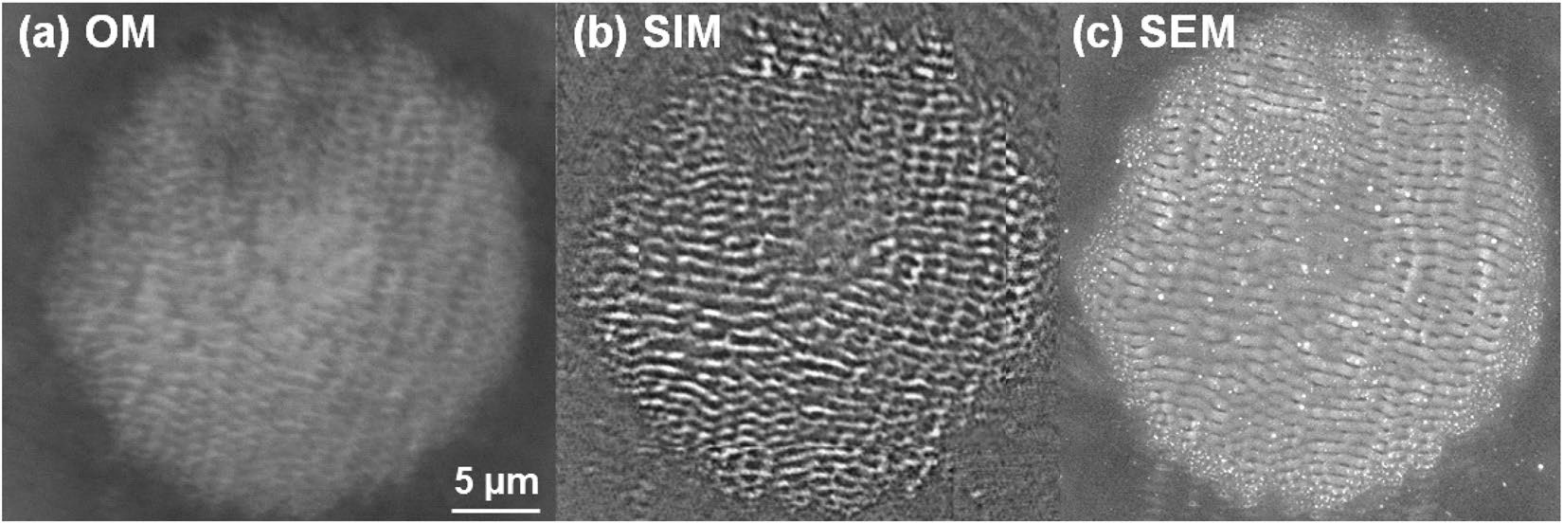
Visualisation of sub-wavelength ripples. LIPSS on a Zr-based BMG surface created after N = 5 ultrafast laser pulses using a peak fluence of 0.3 J/cm^2^. The same pattern is visualized by a range of imaging techniques. (**a**) Impact zone observed using a conventional bright field optical microscope (OM) in reflection mode. (**b**) Impact zone observed *in*-*situ* using SIM based technique during structuring (see text for details). (**c**) Impact observed *ex*-*situ* using a SEM technique.

The results of laser irradiation on the Zr-based metallic glass sample are depicted in Fig. 1(a–c) using different microscopy techniques including standard optical reflection microscopy (Fig. 1(a), OM), structured illumination microscopy with contrast enhancement (Fig. 1(b), SIM) and scanning electron microscopy (Fig. 1(c), SEM). The figure shows clearly the potential of structured illumination microscopy to resolve topography details with high resolution, matching in this type of observations the information provided by SEM. We will then apply the SIM technique for *in*-*situ* observation of ripple development on specific sites with the number of pulses incident on the same spot. Various fluence regimes are used, unveiling explicit behaviors of LIPSS formations.

#### Fluence-dependent ripple evolution

**Figure 2:**
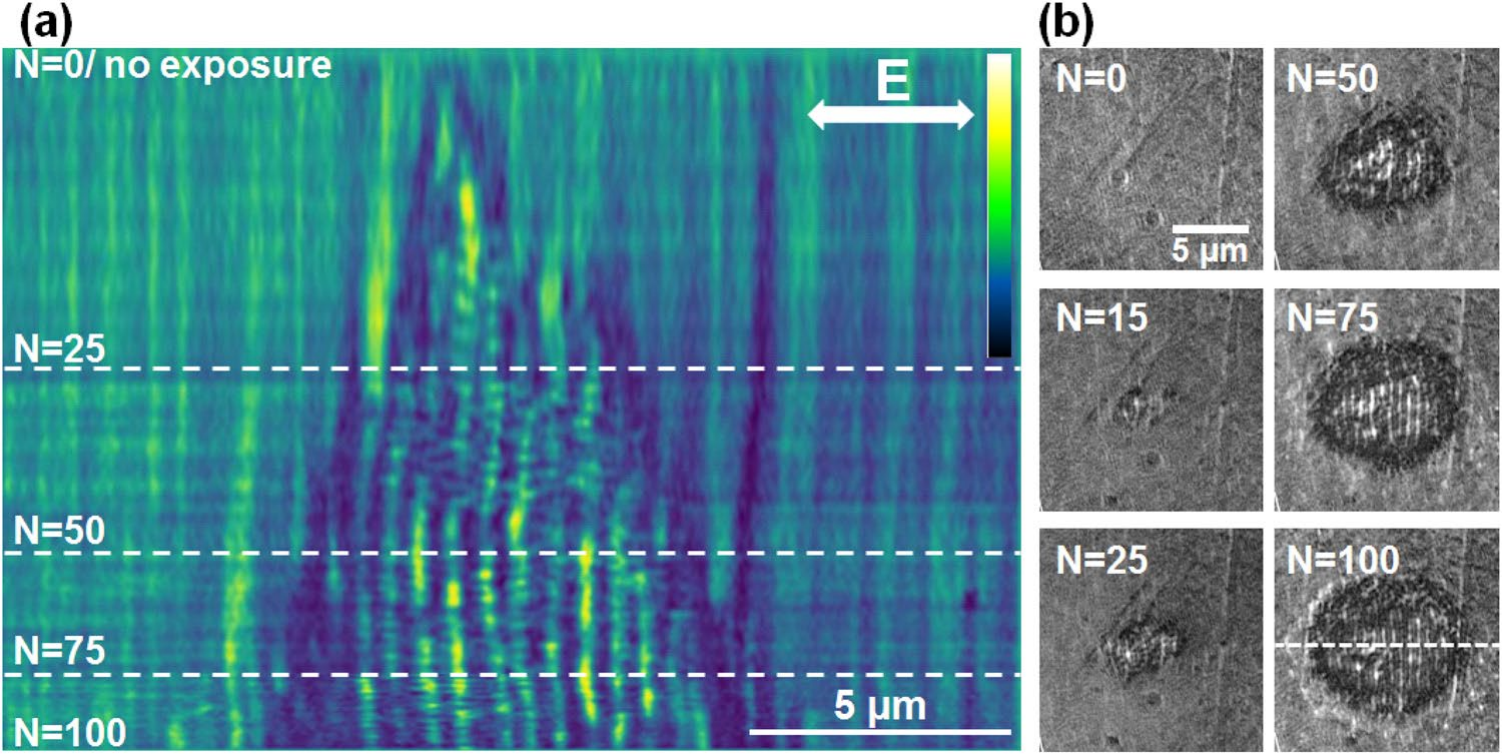
Evolution on LSFL formation on Zr-BMG in the low fluence regime (peak fluence of 0.09 J/cm^2^). (**a**) Evolution of a section profile with the number of pulses *N* (false colors). Sectional cuts made on LIPSS images are concatenated together for each *N*. The LIPSS pattern is visible after a relatively high number of pulses. Light colors represent zones of higher elevation. (**b**) Samples of the original SIM images. The direction of the electric field and the position of the sectioning line are given on the figures.

**Figure 3:**
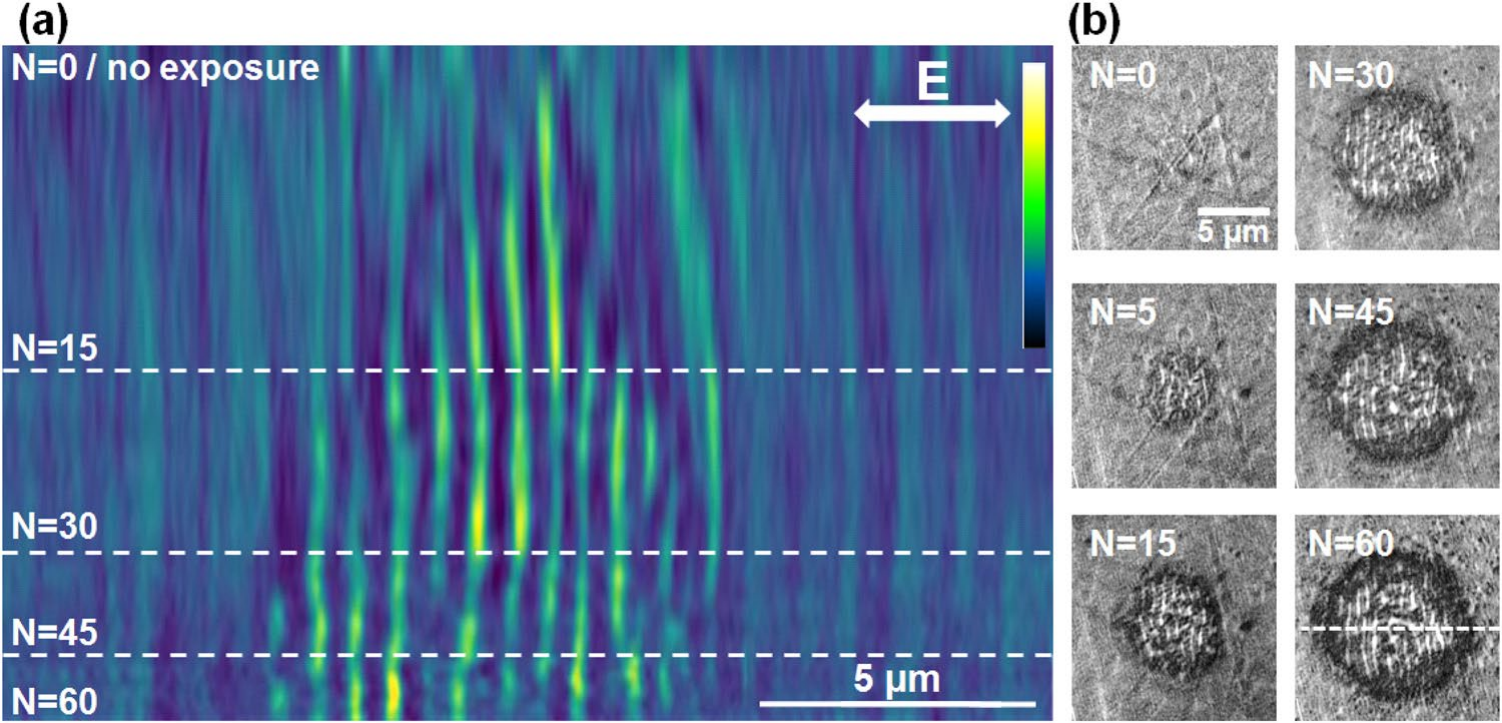
Evolution on LIPSS formation on Zr-BMG in the moderate fluence regime (peak fluence of 0.15 J/cm^2^). (**a**) Evolution of a section profile with the number of pulses *N* (false colors). Sectional cuts made on LIPSS images are concatenated together for each *N*. The LIPSS pattern is detectable earlier in the pulse sequence. Topography features appear already from the first pulses and novel features develop laterally. Light colors represent zones of higher elevation. (**b**) Samples of the original SIM images. The direction of the electric field and the position of the sectioning line are given on the figures.

**Figure 4:**
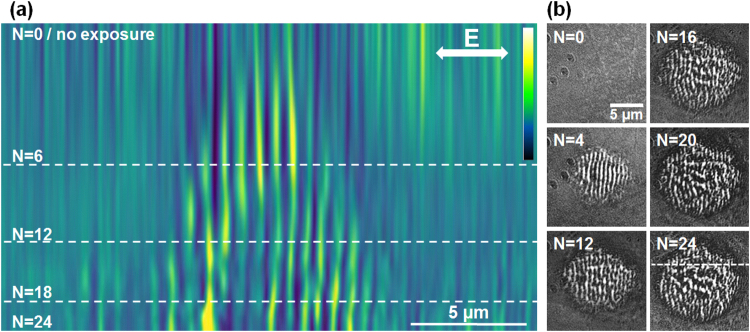
Evolution on LIPSS formation on Zr-BMG in a high fluence regime (peak fluence of 0.31 J/cm^2^). (**a**) Evolution of a section profile with the number of pulses *N* (false colors). Sectional cuts made on LIPSS images are concatenated together for each *N*. At this regime, the rippled pattern appears early in the pulse sequence and is then followed by the formation of an ablation crater. Light colors represent zones of higher elevation. (**b**) Samples of the original SIM images. The direction of the electric field and the position of the sectioning line are given on the figures.

We observe now the gradual development of ripples on a single irradiated site for different incident laser fluences, starting from the threshold vicinity to ablative regimes. The results are shown in Figs 2, 3 and 4. The irradiation sequence commences on the pristine polished surface and stops at the full development of the ripples pattern.

First a low fluence dose (peak fluence of 0.09 J/cm^2^) is used that generates topography ripples on the BMG surface with a period in the range of 600 nm, slightly evolving with the number of incident pulses and fluence. The pattern begins to be observable at a relatively high number of pulses (*N* > 10). This indicates that the modifications after each pulse are minimal, the structures growth is slow, almost erosion-like, and rely heavily on incubation effects. The results of irradiation with increasing number of pulses (*N*) on the same site are given in Fig. 2. The patterns are analyzed in the following way. For successive number of pulses incident on the same site, SIM images were taken and processed. A section is then made across the impact zone and, for each image corresponding to a given *N*, the resulting profiles (ripples relief) are concatenated and displayed in Fig. 2(a) for the whole *N* sequence. Thus the evolution at a specific location can be reconstructed. These profiles are horizontal cuts across a vertical arrangement of ripples and display the regular modulation. This representation gives an indication of the topography development with *N*, where the lateral dimension corresponds to structure periodic topography and the vertical dimension shows the irradiation development with the number of pulses (*N*). Figure 2(b) shows examples of different *N* sequences at the same spot, from where the section was sampled (the position of the sectioning line is given in the figure for the case *N* = 100). If topography features appear after several pulses, they are ill-defined up to *N* = 30. The pattern acquires good visibility after *N* = 50 and shows a remarkable spatial stability with well-defined topographical features preserving their positions for the present experimental conditions. It is though expectable that the period will further decrease for a high number of pulses, a situation not analysed here.

We underline here that the observation of this pattern stability is greatly facilitated by the *in*-*situ* SIM imaging. A similar analysis is applied for a moderate fluence regime (peak value 0.15 J/cm^2^) where rippled patterns appear early in the pulse sequence and resist for a large number of pulses. The results are displayed in Fig. 3(a,b). The pattern develops gradually and grows laterally from pulse to pulse, but preserves constant topography in the affected region from pulse to pulse, with ripple peaks holding constant positions.

The last fluence regime corresponding analysis relates to a relatively high fluence (peak fluence of 0.31 J/cm^2^) where rippled patterns disappear after a certain number of pulses leaving place for an ablation crater. The results are displayed in Fig. 4(a,b). We see that the topography features appear already from the first pulse and the structures are well defined for a low number of pulses. A slightly higher period of 750 nm is found here. With the number of pulses, hydrodynamic movement from the melt is observed culminating with large disordered melting and ablation feature appearing in the crater.

In all cases we observe a continuity of the pattern from pulse to pulse. The features of the profiles are spatially invariant. This is a remarkable outcome, observable only with an *in*-*situ* observation technique with a sufficiently-high resolution, such as the SIM microscopy employed here. The ripple pattern stays stable during the ripples growth with the increasing number of pulses for all fluence ranges. The surface relief stabilizes and becomes reinforced with each pulse, indicating that the topography can lock the pattern distribution. The relief acts as an effective phase and mode-locking mechanism in position and period. This type of stabilizing mechanism is important in developing strategies for patterning large areas, stitching together nanostructured domains and achieving high-quality corrugation upon laser scanning. A feedback mechanism based on both local (plasma evolution and near-field scattering/enhancement) and non-local (far-field scattering and interference) contributions that filters periods and drives the modulation on large domains, growing seamlessly nanostructures was considered essential^22,28^ to develop aligned structures on different periodicity scales, a mechanism which was suggested already in the early studies^29^. The potential origin and the action range of such mechanism are discussed below from an electromagnetic perspective.

### Scattering, interference, and light trapping

The experimental behavior is analyzed first from an electromagnetic perspective. The electromagnetic approach described in the *Methods* section includes an electromagnetic scattering model associated with a straightforward ablation model reflecting the local fluence dependencies and the deposited energy via evanescent components. With this simplified approach we simulate the growth of a specific laser-induced relief. We recall that for metal surface nanostructuring, two different scales of LIPSS formation are usually reported. They correspond to scattering events implying local field enhancement and near-field interactions at roughness features, referred to as high spatial frequency LIPSS (HSFL), and superposition of far-field scattered fields on these roughness centers with incident/refracted fields, referred to as low spatial frequency LIPSS (LSFL)^30^. Let us now concentrate on the most regular ripples pattern, the LSFL.

**Figure 5:**
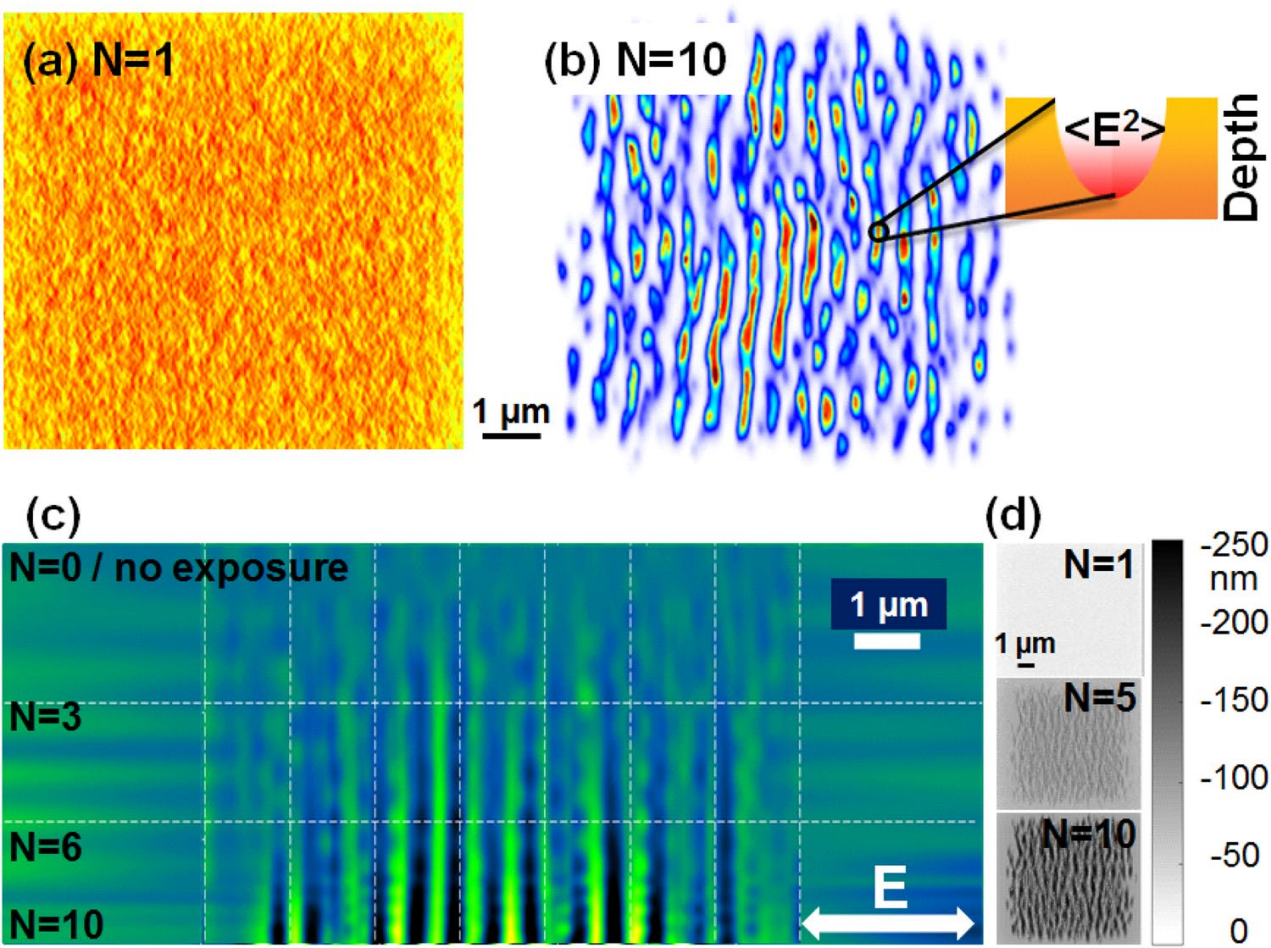
Electromagnetic field computations. (**a**) Simulated energy deposition pattern on Zr-BMG after exposure to a single fs laser pulse on a rough surface. A surface model is used where roughness is estimated via a distribution of 15 nm size nanoparticles and 50% filling factor. (**b**) Simulated energy deposition pattern on Zr-BMG after exposure to *N* = 10 laser pulses. We observe the onset of a clear regular pattern with a periodicity below the wavelength. The insert indicates preferential light trapping and localization at surface depressions. (**c**) Evolution on a sectional topography profile (line section of the surface relief) with the number of pulses. (**d**) Typical computed 2D surface topography profiles for an increasing sequence of incoming pulses.

*Scattering and interference on rough surfaces*. If an optical influence was early recognized^21^ for ripple formation, a comprehensive electromagnetic theory associated with roughness features appeared almost two decades latter^31^. Sipe *et al*.^31^ indicated that a rough surface induces inhomogeneous absorption, where particular spatial frequencies are being preferentially absorbed and imprinted in the material. Thus, the numerical description of the field distribution on surfaces with given topographies is becoming important to evaluate the experimental observations. Following the Sipe analytical approach developed for predicting for which Fourier components the rough surface will concentrate the incident laser energy, we have first simulated a statistical rough surface defining the “selvedge region” where modulation and field enhancement will occur. A typical example of a spatial pattern of deposited energy on a randomly rough surface is given in Fig. 5(a) showing that the energy distribution becomes structured and spatially inhomogeneous. The initial roughness is assimilated to a distribution of 15 nm size nanoparticles and a filling factor of 50%. This distribution was chosen to optimize the field modulation contrast for the sake of visibility, on the expense on a slight reduction of period. The period can be adjusted via the particles size and spacing^32^. The physical origin of the initial roughness can be intrinsic (polishing effects) or extrinsic (laser-induced). The latter is most probably the consequence of a laser-induced nucleation or spallation process at fluences around the melting threshold, process that exhibits a statistical dependence of an Arhenius-like activation energy. A first laser pulse interacting with an initially flat surface close to ablation threshold nucleates solid-liquid transitions^17,33^ at specific “weak” sites, triggering subsurface voids formation, swelling or hydrodynamic instabilities^34^. This randomly modifies the topography of the surface seen by a second pulse, for which the light coupling will be more complex. The coupling complexity involves polarization and scattering effects on the roughness centers, as well as interaction with the incoming field, defining a surface wave pattern^35^. From the near- and far-field interaction of scattered and incident wavelets different distributions of evanescent fields emerge, imprinting their pattern on surfaces, where regular features may appear. In other words, the first step is the appearance of an interference pattern containing the beginning of regular features. For LSFL, with periodicities close to the incident wavelength, the driving field represents the interference between scattered light and the incident wavefront^30^.

*The corrugation mechanism and light trapping*. To describe evolving surface topographies, the electromagnetic simulation is coupled to an ablation model, whenever the threshold is surpassed by the local fluence. In the present model the inter-pulse feedback mechanism was simplified using an holographic ablation model^36^, where the material numerical domains (cells) absorbing more energy were considered to be removed, being replaced by air medium in our simulation. This way, both concentration and amplitude of roughness can evolve during a multipulse simulation. Using the absorption energy map with local concentration of energy driven by interference and near-field enhancement^27^, the application of an ablation model creates an evolving surface topography with elongated surface depressions at the place of energy absorption peaks. Considering the ablation after each pulse, the topography after *N* irradiation events can be reconstituted. Figure 5(b) represents a typical energy deposition pattern upon normal incidence on an initially rough surface, showing the relative value of the electromagnetic energy absorption on the irradiated surface after *N* = 10 absorption-removal cycles. With increasing number of pulses, the order imposes itself with clear ordered patterns. It is noteworthy to mention that a second process occurs in addition to the interference pattern. Local surface depressions of certain depth trap preferentially the light energy, as indicated in the inset in Fig. 5(b). The region between crests is becoming more hollow, developing the ripple contrast pattern at fixed locations. With the evolution of the profile the field distribution becomes potentially more complex, with the appearance of additional Fourier components in its spatial spectrum.

*The evolution of the topography*. We follow now the evolution of the topography contrast upon the arrival of a successive number of pulses. The gradual exposure with increasing number of pulses acquires a structural profile. Using a section cut in the simulated 2D surface profile and concatenating the sequences as before, similar to the experimental situation, Fig. 5(c) retraces the evolution of the surface profile with the number of pulses. Each profile is derived from sectioning computed surface topography charts exposed to a varying number of pulses given in Fig. 5(d). The single site condition is ensured by adopting the same initial surface for each simulated *N*–case. From the profile evolution in Fig. 5(c) we observed a similar representation to that derived from the experimental results in Figs 2, 3 and 4. The simulation clearly shows that, within the removal feedback assumption here, the location of maximal energy absorption does not evolve from pulse to pulse. This strongly corroborates the phase-locked profile amplification scenario reported above based on a scattering and interference effect creating the periodicity and strong light trapping into surface depressions, reinforcing the contrast. This indicates that the main physical features are well grasped by the model, supporting the physical mechanisms proposed above. If the corrugation effect related to pulse-to-pulse evolution can involve a more complex physical picture relying on local ablation, growth, or lateral diffusion of the resulting pattern via liquid displacement, depending on the thermomechanical conditions, the main features of the stabilising feedback are essentially related to interference and potential light trapping.

### Stabilizing patterns on random roughness distribution

We have defined above a first type of optical feedback related to a corrugated topography based on the interference of scattered wavelets with the incident field and light trapping into the corrugated profile. This was facilitated by super-resolved observations enabling to follow *in*-*situ* the gradual development of ripples on a unique irradiated site. A unique site means explicitly a single initial distribution of scattering centers and a singular ablation path, which deterministically constraints the corrugation. Following a scattering model with local and de-localized actions, we indicate that the corrugation defines the scattered pattern and field accumulation regions.

**Figure 6:**
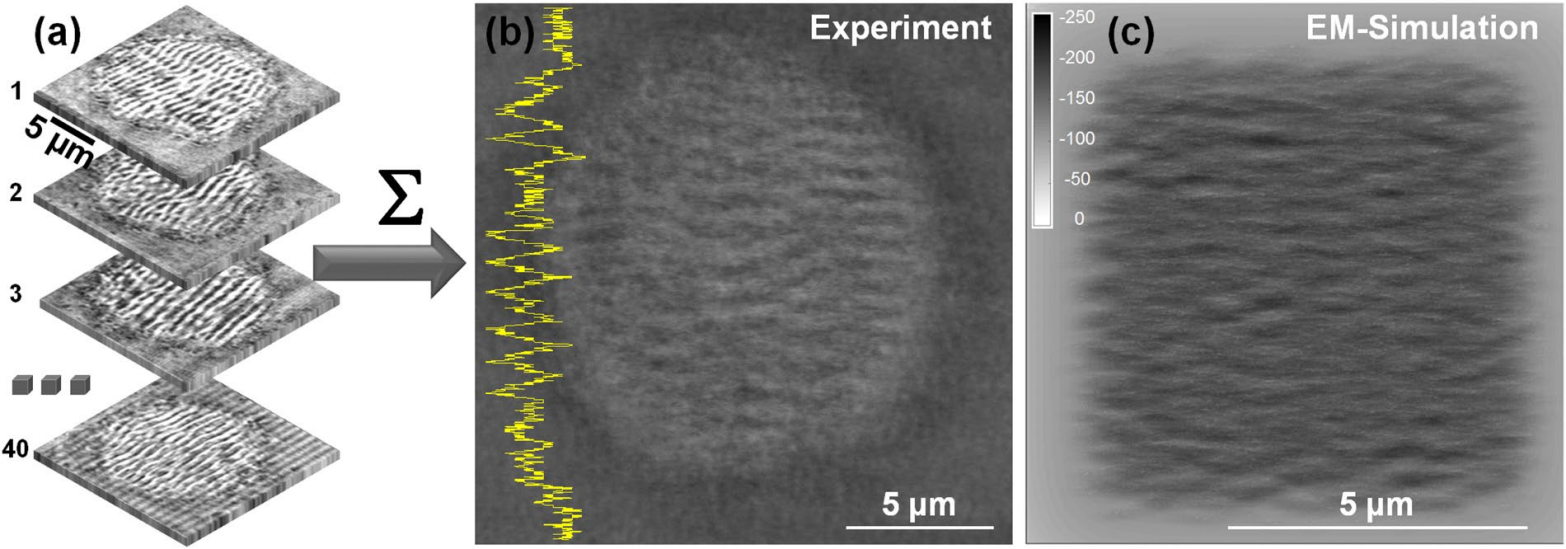
Statistics on random sites. (**a**) Individual images (40) of rippled sites irradiated on various different surface zones of the sample are summed up and averaged. (**b**) The mean result of the images in (**a**) showing a visible corrugation pattern in the sum of the various irradiated zones, suggesting a quasi-stable pattern in-spite of different initial roughness conditions. A line section is equally given, emphasising spatial modulation. (**c**) Electromagnetic calculations showing the average pattern obtained by summing up 16 computed images initiated from a corresponding set of random initial distributions. The average of random corrugation patterns would give an uniform gray level distribution.

The question that appears now is: will a statistical ensemble of different initial sites with *apriori* random roughness distributions affect the pattern arrangement within the laser spot? The *in*-*situ* method allows to compare the irradiation results on different zones on the surface. We have performed the experiment for *N* = 4 laser pulses per site and 40 different initial zones on the sample surface. After acquiring all images, where the relative alignment is ensured by the observation method, we add the images and observe the average result. The procedure is schematically described in Fig. 6(a) and the averaging result is given in Fig. 6(b). The result preserves clearly a periodic character indicating a common absolute pattern of the LIPSS despite the random character of features on the irradiation sites, i.e. the initial surface inhomogeneities. The average of random corrugation patterns before irradiation would have given an uniform gray level distribution. The results is confirmed by performing random image correlations, with the average shift of correlation peak (a measure of the spatial phase) being smaller that Λ/4, Λ being the average pattern period. We have equally performed using the electromagnetic model described above simulations starting from several random initial distributions and summed up the results. A total of 16 simulated profile charts for *N* = 10 starting from initial random roughness functions were calculated and summed up in Fig. 6(c). We equally observe (as indicated in Fig. 6(c)) a permanent corrugation despite the initial random character of the distribution of scattering centers. The reason for this behavior lies in a subtle effect of the electromagnetic interaction between scattered waves and the incident field. Assuming that the scattered field *E*_*scatt*_ on a surface point (*x*_*s*_, *y*_*s*_) will contain a random phase *ϕ*(*x*_*s*_, *y*_*s*_) compared to the incident field *E*_*inc*_, the modulated energy on the surface is related to the interference pattern ${I}_{int}\sim {E}_{inc}{E}_{scatt}\sim {e}^{i\varphi ({x}_{s},{y}_{s})}$. As the correlation function *ϕ* is the single random variable and that the diffuser center (*x*_*s*_, *y*_*s*_) is *δ*-correlated, it can be shown that the correlation function is independent of the surface properties. This means that the phase of the final pattern will be defined by the phase of the laser electric field but not by the roughness properties. This is what we observe both numerically and experimentally where the initial surface roughness is randomly distributed.

The ensemble of experimental and numerical results indicate that an electromagnetic scattering and interference approach is able to reproduce and explain the observed facts, specifically the pattern stability within the laser spot, delivering at the same time the mechanism of spatial phase locking. A generalized electromagnetic approach as originally proposed by ref.^31^ is therefore a good approximation. A relevant question in this perspective is if the observed facts can equally be explained by a surface plasmon interference^20,37^. Similar as scattered wave interference with the incident field, the plasmon interference (surface plasmon polariton model) would lead in our opinion to a similar conclusion with respect to the spatial arrangement. A detailed inspection of the nature of the created evanescent fields exceeds however the frame of this work and requires detailed analysis of the scattered waves^35^. The present scattering approach has however no explicit requirement to rely on plasmonic excitation (in view to their propagation limitations on rough surfaces^38^) as evanescent fields can be coherently generated by scattering mechanisms, and it thus represents a more general case.

## Methods

### Experimental procedure: structured illumination microscopy

**Figure 7:**
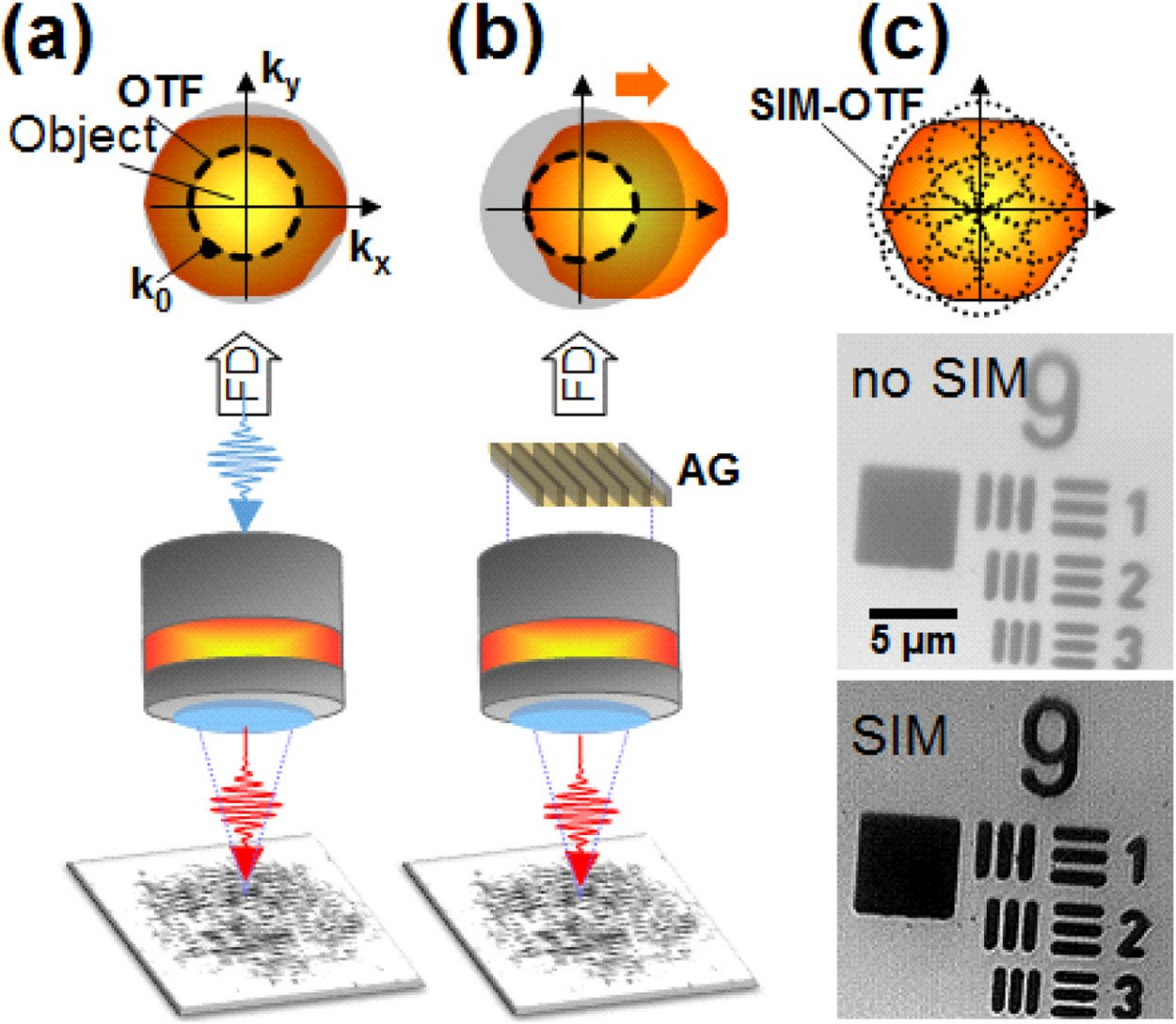
Experimental set-up for *in*-*situ* investigations of LIPSS growth using SIM. The fs laser beam in red is injected into the light path of the microscope. (**a**) Bright-field epi-illumination microscopy whose OTF is limited by the Rayleigh criterion *k*_0_. (**b**) SIM microscopy where a sinusoidal amplitude grating (AG) is imaged onto the sample surface. This sinusoidal modulation yields a frequency shift in the FD allowing to observe a fraction of the higher object spatial frequencies. (**c**) By recording images for an ensemble of AG positions, the SIM permits to achieve an extension of the OTF as illustrated by the pictures with and without SIM of the USAF target. The achieved lateral resolution is 150 nm.

The irradiation system presented in Fig. 7 uses a common path excitation and observation module. The SIM is integrated in the irradiation setup following the same path as the processing ultrafast laser beam. This allows the *in*-*situ* observation of the nanostructures during the laser structuring events.

Structured illumination microscopy (SIM) is a full-field super resolution optical technique enabling super resolution using a sinusoidal modulation on the light projected over the sample. It allows to improve by a factor of two the standard optical resolution defined by the imaging optics. Conceptually, the structured illumination is equivalent to an artificial augmentation of the observation numerical aperture, collecting and adding high-spatial frequency information to the image that is missing in standard optical microscopy. This type of microscopy was first demonstrated on fluorescent polystyrene micro-spheres^39^ and is adapted here to investigate the LIPSS growth *in*-*situ* on surfaces. The SIM concept is briefly recalled here. A regular optical microscope has a resolution limit mainly related to the objective’s numerical aperture (NA), leading to a cut-off in the spatial frequency domain. Thus, the microscope acts as a low-pass filter whose cut-off frequency limits the optical transfer function (OTF) of the microscope. For simplicity, this can be represented as a limiting circle in the Fourier Domain (FD) (see Fig. 7(a)). Any high frequency information of the object outside this circle is lost when imaging through the microscope. With SIM, a sinusoidal modulation of the illuminating light is performed using e.g. an amplitude grating. This modulation acts as a frequency shift of the object in the Fourier domain. If the modulation period equals the optical microscope diffraction limit, the object high spatial frequencies can be moved within the microscope’s OTF (see Fig. 7(b)). By recording several images with different angles and translations of the grating, a super resolution image can be reconstructed (see Fig. 7(c)) with a resolution overcoming the diffraction limit by a factor of two. The technical description of the microscopy setup is provided below. A LED source is used for the setup with a central wavelength of *λ* = 405 nm and a bandwidth of 13 nm; the light is filtered by a diffuser and collimated, achieving an uniform incoherent epi-illumination. The objective NA is 0.9, yielding a Rayleigh criterion of *k*_*o*_ = 275 nm in bright-field mode. The structured illumination light projection is performed using an amplitude grating. The grating used in our experiment is a Ronchi ruling with a period of 60 lines/mm. The grating is mounted on rotation and translation stages, such as its position and rotation can be adjusted for data gathering for the spectral domain OTF extension process. The grating can be projected onto the back focal plane of the microscope objective by means of a beam splitter. The grating magnification is given by imaging system, achieving a period of the fringes on the samples of 300 nm, which is purposely very close to *k*_*o*_. Using this experimental apparatus, the attained SIM resolution is 150 nm.

To enhance the figure contrast a image treatment procedure was applied. Periodic nanostructures contrast was enhanced using a band pass filter in the spatial frequency domain. The filter was designed to cut off the information with spatial frequency not corresponding to the nanostructures; once the filtered image was processed, an average of the filtered nanostructured image and the background image was done.

### Modeling approach: electromagnetic model

An electromagnetic model in 3D geometry^30,40^ is used to calculate field irradiation patterns. The light pattern is assumed to result from a superposition of scattered wavelets from individual scattering centers with the incoming field. The inhomogeneous energy deposition below the material rough surface is computed based on 3D Maxwell’s equations solved within the Finite-Difference Time-Domain (FDTD) method depicted in ref.^30^. To construct a rough surface, the dielectric function of the top layer of the simulated material is described by a two-value function *ε*_*r*_(*x*, *y*). That is, *ε*_*r*_(*x*, *y*) = *ε*_*BMG*_, for the filled region and *ε*_*r*_(*x*, *y*) = 1 for the unfilled region, where *ε*_*BMG*_ is the dielectric function of the simulated material. The filling factor *F* defined as the mean value of the binary function *f* (*f*(*x*, *y*) = 1 for the filled region and *f*(*x*, *y*) = 0, otherwise) was set to 0.5. Above the roughness layer, the source consisting of the electric field component *E*_*x*_ is injected into the computation grid as a soft source. The pulse duration was fixed to *τ* = 30 fs to ensure a steady state solution. The pulse travels downward towards the material, impinging the surface at normal incidence. To maximize the interaction area between the incident pulse and the rough surface, the fluence distribution was a super-Gaussian type of order 7. To simulate the increasing dose a simplified ablation model is used implying material removal (15 nm rate, equivalent to 1 cell) where local fields exceed the ablation threshold. The energy absorbed in the selvedge zone is calculated in the medium assuming non-evolving optical properties, BMG *ε* = −6 + 15.16*i* and air *ε* = 1. The dielectric function of the BMG does not vary significantly during the excitation period as measured by time-resolved ellipsometry^32^, particularly due to its multicomponent nature resulting in a large electronic density of states.

## Conclusion

In conclusion, we have developed a super-resolved optical method for *in*-*situ* observation of laser nanostructuring processes relying on structured light illumination microscopy in a common path with the irradiation beam steering. Based on the achieved resolution and optical contrast, we have monitored the ripple formation on metallic glasses with the accumulated dose in various fluence regimes involving slow, gradual erosion-like changes at low fluence as well as the apparition of molten phases at higher fluences. We have established the action of a spatial phase-locking mechanism in the generation of laser-induced periodic structures, originating from the coherent interaction of incoming and scattered waves. This involves an implicit effect where the initial field repartition on a rough surface with corrugation in a periodic pattern will fix the light distribution for the incoming pulse. This corrugation effect acts as an effective mode-locking mechanism, filtering the resulting periodicity. The effect relies on field scattering, interference and light enforcement in surface topographical depressions. Additionally, a more subtle effect can spatially fix the pattern even on surfaces with random roughness and topographies. This is based on the fact that the superposition of scattered wavelets are self-referenced on the driving field, fixing their far-field interaction with the incoming incident light front. The phase of the pattern fixing its relative position is thus imposed by the incoming field. The highly-resolved *in*-*situ* observation allows thus novel essential features into the development of laser periodic nanostructures, with feedback that corrects and stabilizes period and shift. The understanding of laser-driven self-arrangement of surfaces and the driving feedback will contribute to a high level of process control in emerging laser large-scale nanostructuring techniques.
